# Effect of Eel Biscuit Supplementation on Height of Children with Stunting Aged 36–60 Months: A Pilot Study

**DOI:** 10.1155/2020/2984728

**Published:** 2020-05-24

**Authors:** Dewi M. D. Herawati, Siti N. Asiyah, Siska Wiramihardja, Shifa Fauzia, Deni K. Sunjaya

**Affiliations:** ^1^Department of Public Health, Division of Medical Nutrition, Faculty of Medicine, Universitas Padjadjaran, Bandung, Indonesia; ^2^Magister of Public Health Programme, Faculty of Medicine, Universitas Padjadjaran, Bandung, Indonesia; ^3^Magister of Basic Medical Science Programme, Faculty of Medicine, Universitas Padjadjaran, Bandung, Indonesia

## Abstract

**Background:**

Stunting is a major health problem in developing countries. Animal-based supplements can increase the height of children with stunting. This study was aimed at determining the effect of eel biscuit supplementation on increase in the height of children with stunting aged 36–60 months.

**Methods:**

A pilot study with pretest-post-test design. The study was conducted in two villages in the Priangan Region, West Java Province, Indonesia. The participants were divided into two groups: intervention group (10 supplemented eel biscuit pieces) and control group (biscuits from the government's biscuit programme). A total of 56 children aged 36–60 months with the height-for-age *z*-score of <−2 SD were recruited voluntarily for sampling.

**Results:**

The initial height-for-age *z*-score of the intervention group was −3.45 SD and that of the control group was −3.11 SD. After 3 months of supplemented eel biscuit consumption, the height-for-age *z*-score of the intervention group changed to −2.52 SD and that of the control group changed to −2.51 SD. The average shift of the height-for-age *z*-score after 3 months of supplemented eel biscuit consumption was 0.93 SD in the intervention group and 0.6 SD in the control group. There were significant differences in delta and percent increase in the height-for-age *z*-score between both groups.

**Conclusions:**

Consumption of supplemented eel biscuits for 3 months increased the height-for-age *z*-score of children with stunting aged 36–60 months by 0.93 SD.

## 1. Introduction

Stunting is one of the main health problems in developing countries and is considered a marker of delayed growth. Indonesia, a country in Southeast Asia, has the highest prevalence of stunting (36.4%), followed by Philippines (30.3%), Myanmar (29.2%), Malaysia (17.7%), and Thailand (16.3%) [1]. Several studies have demonstrated that the effects of stunting occur in both short- and long-term forms including linear growth disorders, decreased cognitive function, impaired immune function, and metabolic disorders, which ultimately increase the risk of degenerative diseases such as obesity, hypertension, and diabetes mellitus [2]. Hoffman et al. demonstrated that fat oxidation is common in children with stunting, with there being a tendency of fat storage in the abdominal area. This often predicts the occurrence of metabolic diseases [3, 4].

A child is diagnosed with stunting if his/her height measurement is lower than the standard height, which is set according to his/her age (low height-for-age value). According to the World Health Organization (WHO), stunting is defined as a height-for-age *z*-score (HAZ) of below −2 standard deviations (below −2 SD) on the WHO chart of growth standards based on sex [5]. Height is one of the important parameters of health status. Although a toddler's height in the first 2 years of life is determined by the mother's health during pregnancy and intrauterine nutrition and growth, height also reflects optimal metabolic adaptation, organ maturation, and risk of disease in adulthood [6].

According to Onyango et al. and Arimond and Ruel, the diversity in food consumed is related to growth, i.e., consumption of more varieties of food ensures better linear growth in infants [7, 8]. One of the causes of stunting is a lack of long-term macronutrients and micronutrients. One of the main functions of macronutrients is as a source of energy. Energy shortage causes various disorders including growth disorders [9]. Energy is required for all metabolic activities; therefore, the mechanism underlying disturbances in the body due to a lack of energy can be highly complex. One of the important macronutrients is protein, with its quality being particularly important. This is based on the study by Uauy et al., who stated that high-quality proteins in complementary foods have been demonstrated to be effective in increasing growth [10]. Similarly, in a study by Ghosh et al. in developing countries, there was an inverse relationship between stunting occurrence and protein quality [11]. Protein quality is imperative because amino acids, as the main component of proteins, also have important functions in growth. Insufficient amino acids in the body can cause disrupt protein synthesis and ultimately affect body activities including growth. For example, arginine is one of the functional amino acids known to have a direct role in growth. It is associated with growth hormone (GH) release and somatostatin inhibition [12]. By contrast, lysine affects growth through its role in carnitine biosynthesis, calcium absorption, and collagen biosynthesis [13].

Furthermore, important micronutrients for growth include zinc, vitamin A, iron, and calcium. For example, Gibson et al. and Tabatadze et al. demonstrated that zinc levels in the serum and hair were lower in children with stunting than in those without stunting [14, 15]. Conversely, Brown et al. demonstrated that zinc supplementation can increase body weight and linear growth significantly [16]. Other studies support the importance of vitamin A in growth. For example, Abedi et al. indicated that in India, there was a strong relationship between stunting occurrence and inadequate vitamin A intake [17].

Amino acids (such as arginine and lysine), calcium, zinc, iron, and vitamin A are found in eels. The bicolor eel is a fish native to Indonesia. It is a marine catadromous fish that is spawned in seawater, lives in fresh water (rivers and lakes) in its adulthood, and then returns to the deep sea to reproduce.

Nutritional supplements are crucial to improvement in the stunting phenotype. These supplements should be designed, and their administration should be monitored carefully to prevent exposing children with growth issues to the risk of being overweight instead [18]. The Ministry of Health of Indonesia has initiated a biscuit programme to help alleviate child malnutrition. These biscuits have been distributed to malnourished children aged below 5 years. However, this program does not include a biscuit intended for children with stunting. Therefore, because these fish contain important nutrients for growth, the researchers created snacks in the form of biscuits supplemented with eels, targeted for children with stunting. The purpose of this study was to analyse the effect of eel biscuit supplementation on the height of children with stunting aged 36–60 months.

## 2. Materials and Methods

### 2.1. Study Design and Setting

West Java, the most populated province in Indonesia, is divided into four regions. The Priangan Region has the most cases of stunting, with a prevalence of approximately 43%. This study was conducted in the Cibungur Village and the Pangadegan Village, which are rural areas. We recruited all children aged 36–60 months from the two villages to identify children with stunting, with a total of 290 children identified. We conducted physical examination and nutritional assessments at the Integrated Health Post. A primary health centre doctor conducted physical examination with the assistance of village midwifes. Based on the results of anthropometric measurements and physical examinations, as well as inclusion criteria, the children were recruited as participants in the study. We selected children who had short or very short height according to the WHO criteria (<−2 SD) and were willing to eat eel biscuits for 3 months. Children with chronic disease, congenital disease, or severe acute malnutrition were excluded. Participants were selected using simple random sampling. Sample size was determined for the comparison of two proportions with a power of 80% and a significance level of 5%.

This pilot study included a pretest-post-test design. The participants were divided into two groups, each with 28 participants: those in the intervention group consumed eel biscuits and those in the control group consumed biscuits from the government programme (without the eel formula). All participants consumed 10 pieces of biscuit per day (5 in the morning and 5 in the afternoon) for 3 months. During the study, two children from the intervention group and one from the control group dropped out.

We considered the possibility of biscuit exchange among participants if they are too close to each other. To avoid this, all participants in the intervention group were recruited from one village and those in the control group were from the other village.

### 2.2. Ethical Approval

The purpose of the study was explained verbally to the parents of the selected children who then provided written approval, witnessed by health officers. The study was conducted according to the Declaration of Helsinki and received ethical clearance from the Ethics Committee of the Faculty of Medicine, UNPAD, no. 28/UN6.C1.3.2/KEPK/PN/2017.

### 2.3. Data Collection and Measurements

All participants were subjected to nutritional assessment that included anthropometric measurements and dietary assessments. Anthropometric measurements of height-for-age were conducted before intervention and in the first, second, and third months after intervention, which were performed by trained and experienced field nutritionist officers. Height was measured using a rigid height board (Seca 417) with an accuracy of 0.1 cm. HAZ was calculated using the WHO growth curve. A 24-hour dietary recall was conducted before and after the intervention by the research team assisted by nutritionists, and food records were done after the intervention by the mothers of the participants, who had been trained by the research team prior to the intervention. This was performed to determine whether there was a change in food intake among the participants. Health cadres, one per five participants, randomly monitored, supervised, and reported about biscuit consumption daily. The health cadres collected food records weekly from the houses of the participants.

### 2.4. Biscuit Supplementation

Nutritional supplements are foods that are specifically formulated to provide additional energy and nutrients that are lacking or available only in limited quantities in daily food [19]. Supplementation can be administered in various forms such as biscuits. Biscuits are typically liked by all age groups, particularly toddlers. Therefore, they can be given to help increase toddler's nutritional intake. These biscuits must have good nutritional content and meet required quality standards. In particular, they must maintain health, improve the immune system, and facilitate recovery.

To achieve a quality that meets standards and possesses enhanced functional value, the biscuit composition should contain various ingredients that have good nutritional value. Animal-based food sources can be used for this purpose. Besides high energy and protein content, animal-based food sources tend to have complete micronutrients. In this respect, they may often be superior to plant-based food sources [20].

Eel is an animal-based food source that can be used as supplementary food for toddlers. However, to extend the shelf life of biscuits and increase their usefulness, the fish can be added to flour. This combination can then be used as a substitute for wheat flour during biscuit preparation. Using fish flour in biscuits for toddlers can increase the functional value of the biscuits. One of the advantages of fish is its high protein content. Moreover, their balance of amino acids is close to human needs. Fish flour substitutes (and soybean isolates) increase the protein content of biscuits [21]. The advantage of these eel biscuits is that they have good macronutrient and micronutrient contents, which is particularly beneficial in promoting growth in children. The biscuits used in this study contained eel bone flour and *cilembu* sweet potato flour.

Proximate analysis findings and nutritional facts (Table 1) obtained at the Saraswanti Bogor Laboratory as well as metal contamination and microbiological findings met the quality and safety standards for biscuit foods according to the Indonesia National Standard.

### 2.5. Statistical Analysis

Univariate analysis of demographic characteristics of the participants' parents was performed using the unpaired *t*-test and the chi-squared test. Bivariate analysis for energy intake was performed using the unpaired *t*-test if data were normally distributed. Otherwise, the Mann–Whitney test was performed. Meanwhile, protein intake was analysed using the paired *t*-test, and HAZ was calculated for both groups using the unpaired *t*-test and the paired *t*-test. Statistical significance level (*p*) was set at <0.05.

## 3. Results and Discussion

### 3.1. Results

Analysis of demographic characteristics indicated that the age, qualifications, and occupations of the participants' parents were not significantly different between the two groups (Table 2). Most parents had low education levels, with fathers working as labourers and mothers being unemployed. This may explain the source of stunting among the participants.

Table 3 indicates that the preintervention mean energy intake was higher in the control group (870 kcal) than in the intervention group (845 kcal), similar to the postintervention mean energy intake (1392 vs. 1364 kcal). Comparison of preintervention and postintervention mean energy intake indicated that the increase in the mean energy intake was higher in the intervention group (546 kcal) than in the control group (521 kcal), but the difference was not significant.

As shown in Table 4, the increase in protein intake after intervention was lower in the intervention group (0.36 g) than in the control group (0.92 g), but the difference was not significant.

Compliance with biscuit consumption was higher in the intervention group than in the control group (Table 5). The majority of the participants in the control group only consumed 4–7 biscuits per day.

**Figure 1:**
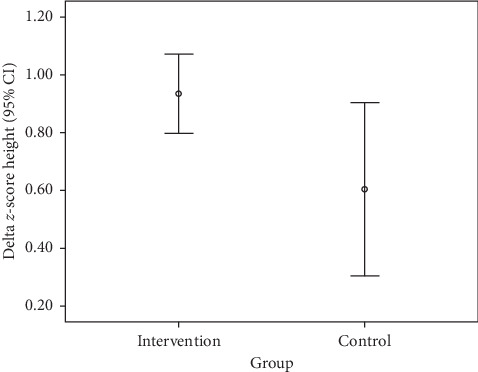
Changes in delta and percent of the height-for-age *z*-score in both groups.

During the 3 months of biscuit consumption, the negativity of HAZ decreased in both groups (i.e., degree of stunting reduced; Table 6). The mean shift in HAZ over the 3 months was 0.93 SD in the intervention group and 0.60 SD in the control group. The difference in the initial HAZ was approximately 0.34 SD (i.e., −3.45 in the intervention groups vs. −3.11 in the control group), but after 3 months of biscuit consumption, the difference has narrowed to 0.01 SD. The difference in delta and percent increase in height between the groups was significant. Eel biscuit supplementation had a higher effect on HAZ in the intervention group than in the control group (Figure 1).

### 3.2. Discussion

The results of this study indicated that biscuit consumption for 3 months improved HAZ in both groups (i.e., the negativity of HAZ decreased in both groups, indicating that the degree of stunting decreased). The postintervention improvement in HAZ was significant in the intervention group compared with the control group. It is possible that if the eel biscuits were consumed for >3 months, HAZ would have been higher in the intervention group than in the control group.

The findings of this study are consistent with those reported by Ramakrishnan et al. They conducted a meta-analysis and indicated that consumption of supplementary foods containing both single and multiple micronutrients is associated with decrease in the degree of stunting, to a certain extent, in children aged <5 years [22].

However, it was also found that with such interventions, it is essential that sufficient time is allowed to observe the beneficial effects. Walker et al. demonstrated that the effects of the provision of nutritional supplementation and psychosocial stimulation in children with stunting could only be seen after 6 months of intervention, whereas increased length, weight, and head circumference could only be seen after 12 months of intervention [23]. Similarly, the effects of the intervention conducted by Zhang et al. to increase the height of children with stunting aged between 6 and 23 months who were given complementary food supplements could not be observed even after 1 year [24]. This may be due to the low carbohydrate and protein contents of complementary foods given to the children although there was an additional 4.0 g protein and fat from soybeans in each sachet. Another possibility is that more time is required to observe its impact on stunting [24].

The eel biscuits had high contents of energy, fat, protein, arginine, lysine, vitamin A, calcium, iron, and zinc. It is possible that these nutritional features of the eel biscuits were responsible for the extra growth of children with stunting. Food energy may be of particular importance because it is required for every metabolic activity and to optimise the use of proteins for growth. Therefore, energy shortage may cause various disorders including growth disorders [9]. The content of arginine and lysine in the eel biscuits may also be beneficial for promoting growth in children. This is supported by the study by Van Vught et al. in Malawi, which indicated that there was a positive correlation between HAZ and amino acid content [25]. In addition, in China, Zhao et al. demonstrated that children with high arginine and lysine intake have better growth [26]. Consistent with these observations, Nuss et al. asserted that a low intake of essential amino acids such as lysine can result in a high risk of stunted growth in children [27]. This is supported by the study by Uauy et al. who demonstrated that low amino acid intake is associated with a high frequency of stunted growth [28].

Other nutrients in the eel biscuits may also be important. The contents of vitamin A and iron are linked to increased growth among children with stunting. Rohner et al. observed that the consumption of fortified foods containing vitamin A and iron can promote growth in children [29]. Vitamin A can affect growth through various mechanisms including its role in gene expression, immune system, and growth factors and in facilitating the activity of enzymes involved in bone remodelling.

The zinc content of the eel biscuits is another micronutrient that possibly played a role in the increase in growth among children [16], particularly in those aged >2 years [30]. Its deficiency has a detrimental effect on growth among children [31]. Zinc affects growth through its role in DNA replication, immune system, appetite stimulation, and GH release; therefore, it is essential for growth and development, immune responses, and cognitive function [32]. Calcium is another mineral that may help explain our findings. It has an important role as a major structural component in bone formation. Its deficiency affects the growth of children [33].

Animal-based food sources such as fish, which are rich in nutrients, can be of value for promoting growth among children. The nutrients in animal-based food sources tend to be more easily absorbed and used by the body than plant-based food sources [19]. A study from China reported that toddlers who were given complementary foods from animal-based sources experienced a greater increase in height than those given only plant-based food sources [34]. According to Michaelsen et al., animal-based foods that are good sources of minerals and proteins are important for the growth of children [35].

This study has certain limitations. The sample size was small, and the duration of intervention (3 months) may not have been long enough. A follow-up study is needed with a larger sample size and longer intervention (>6 months).

## 4. Conclusions

Consumption of eel biscuits for 3 months children with stunting aged between 36 and 60 months is associated with an increase in HAZ. Therefore, the results of this study suggest that these biscuits are of value for helping to reverse stunting. However, a larger study with a longer follow-up is needed.

## Figures and Tables

**Table 1:** Comparison of nutritional content between eel biscuits and control biscuits.

Parameter	Eel biscuits	Control biscuits
Energy (kcal)	**446**	450
Fats (g)	**20.6**	15
Protein (g)	**16.5**	9
Carbohydrate (g)	**50.8**	70
L-arginine (mg)	**4333.93**	—
L-lysine (mg)	**4490.13**	—
Vitamin A (mcg)	**116.95**	—
Zinc (mg/ppm)	**29.85**	—
Iron (mg/ppm)	**38.1**	—
Calcium (mg)	**921.86**	—

**Table 2:** Demographic characteristic of the participant's parents.

Variable	Groups		*p*
	Intervention *n* = 25	Control *n* = 27	
1 Age of father (years)			
x (SD)	34.00 (5.1)	37.37	0.06
Median	34.0	37.0	
Range	25–46	27–60	
2 Age of mother (years)			
x (SD)	30.8 (5.3)	32.22 (6.16)	0.376
Median	31.5	32	
Range	22–39	23–48	
3 Qualification of father			
Elementary school	15 (57.7)	17 (63.0)	0.558
Junior high school	8 (30.8)	9 (33.3)	
Senior high school	3 (11.5)	1 (3.7)	
4 Qualification of mother			
Elementary school	13 (50.0)	14 (51.9)	0.898
Junior high school	9 (34.6)	10 (37.0)	
Senior high school	4 (15.4)	3 (11.1)	
5 Occupation of mother			
Employed	4 (7.7)	4 (14.8)	0.953
Unemployed	22 (92.3)	23 (85.2)	
6 Occupation of father			
Private employees	2 (7.7)	4 (14.8)	0.304
Entrepreneur	9 (34.6)	13 (48.1)	
Labourer	15 (57.7)	10 (37.1)	

**Table 3:** Preintervention and postintervention mean energy intake (calories) in both groups.

Variable	Groups				*p*
	Intervention *n* = 25		Control *n* = 27		
	Preintervention	Postintervention	Preintervention	Postintervention	
Energy intake					
x (SD)	845 (199)	1364 (157)	870 (203)	1392 (207)	
Median	826	1345	847	1418	
Range	476–1304	1088–1670	481–1326	993–1728	
*p*	0.001	0.001	—	—	
Delta (Δ) = postintervention − preintervention					0.884
x (SD)	546 (221)		521 (181)		
Median	524		509		
Range	144–938		174–834		

**Table 4:** Preintervention and postintervention protein intake (grams) in both groups.

Variable	Groups				*p*
	Intervention *n* = 25		Control *n* = 27		
	Preintervention	Postintervention	Preintervention	Postintervention	
Protein intake					
x (SD)	22.7 (7.0)	23.1 (5.1)	22.4 (4.1)	23.3 (4.4)	
Median	21.5	23.0	24.0	23.3	
Range	10–37	16–36	11–29	11–30	
*p*	0.763		0.123		
Delta (Δ) = post − pre					0.695
x (SD)	0.36 (6.4)		0.92 (3.01)		
Median	1.5		1		
Range	(−19)−12		(−5)−6		

**Table 5:** Compliance with biscuit consumption in both groups (%).

Consumption of snacks	Groups	
	Intervention (*n* = 25)	Control (*n* = 27)
Not consumed	—	—
1–3 pieces	—	—
4–5 pieces	—	37%
6–7 pieces	—	33%
8–9 pieces	39%	19%
10 pieces	61%	11%

**Table 6:** Combined mean estimate (standard error) height-for-age *z*-score before intervention, after the first month of intervention, and after the third month of intervention in the study groups.

Time	Groups		Mean difference (95% CI)	*p*
	Intervention	Control		
	(*n* = 25)	(*n* = 27)		
Before intervention	−3.45 (0.13)	−3.11 (0.12)	−0.34 (−0.70; 0.02)	0.065
One month after intervention	−3.07 (0.12)	−2.92 (0.12)	−0.15 (−0.49; 0.19)	0.387
Three months after intervention	−2.52 (0.13)	−2.51 (0.13)	−0.01 (−0.38; 0.37)	0.973
Comparison:				
Before intervention vs. 3 months after intervention	*p* < 0.001 0.93 (0.07)	*p* < 0.001 0.60 (0.14)	0.33 (0.10; 0.66)	0.047
Delta (3 months after intervention − before intervention)	27.82 (2.08)	17.25 (4.13)	10.57 (1.21; 19.93)	0.028

## Data Availability

The data used to support the study are available from the corresponding author upon request.
